# Supplementary figures and images for: Tissue-Specific Landscape of Metabolic Dysregulation during Ageing
Source: Biomolecules. 2021 Feb 7;11(2):235. doi: 10.3390/biom11020235 (PMC7914945; doi:10.3390/biom11020235)

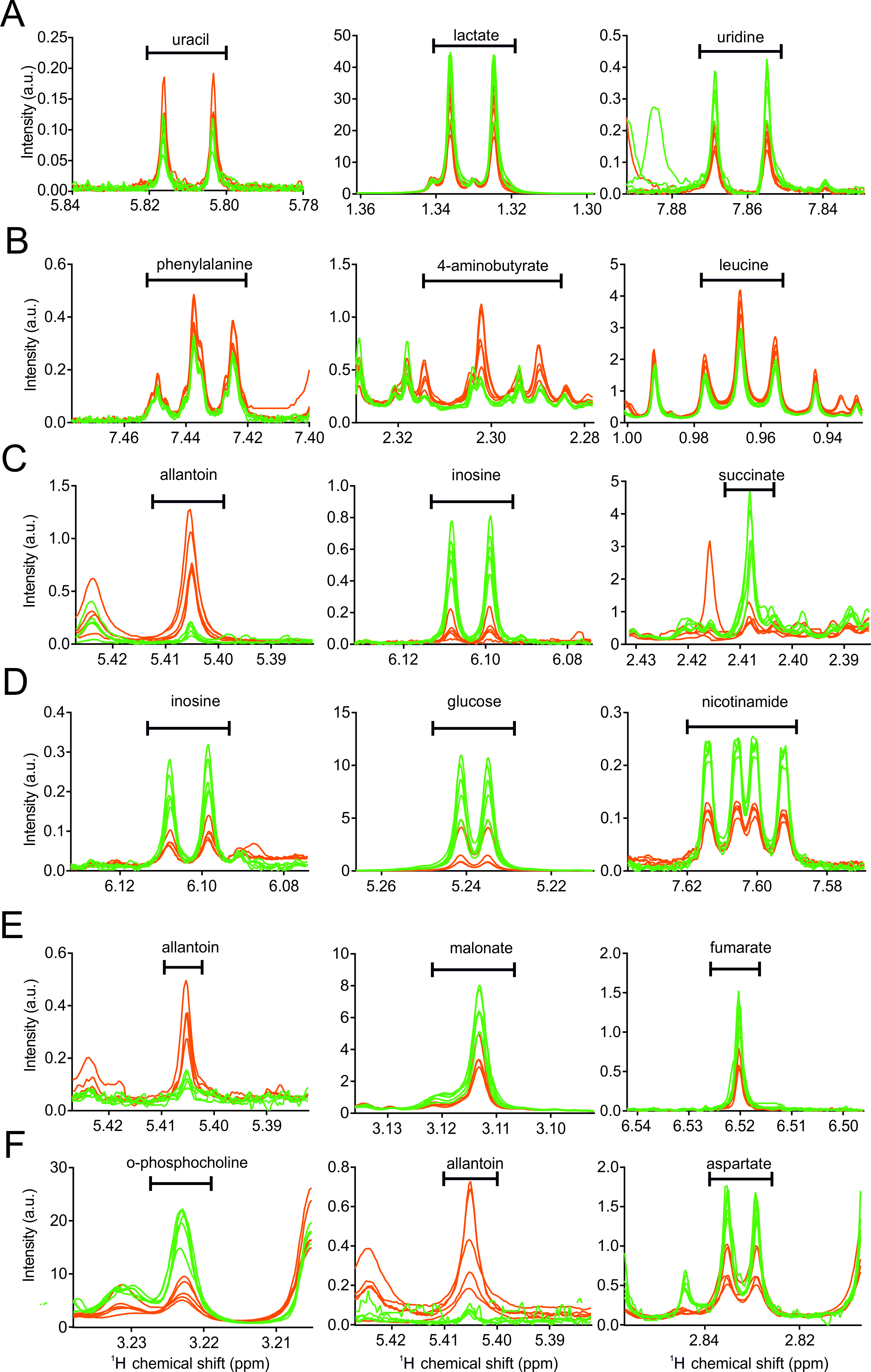

Supplement: Supplementary file 1 [file biomolecules-11-00235-s001.zip › Biomolecules 1047494/Figure S1 metabolitesv5.tif]

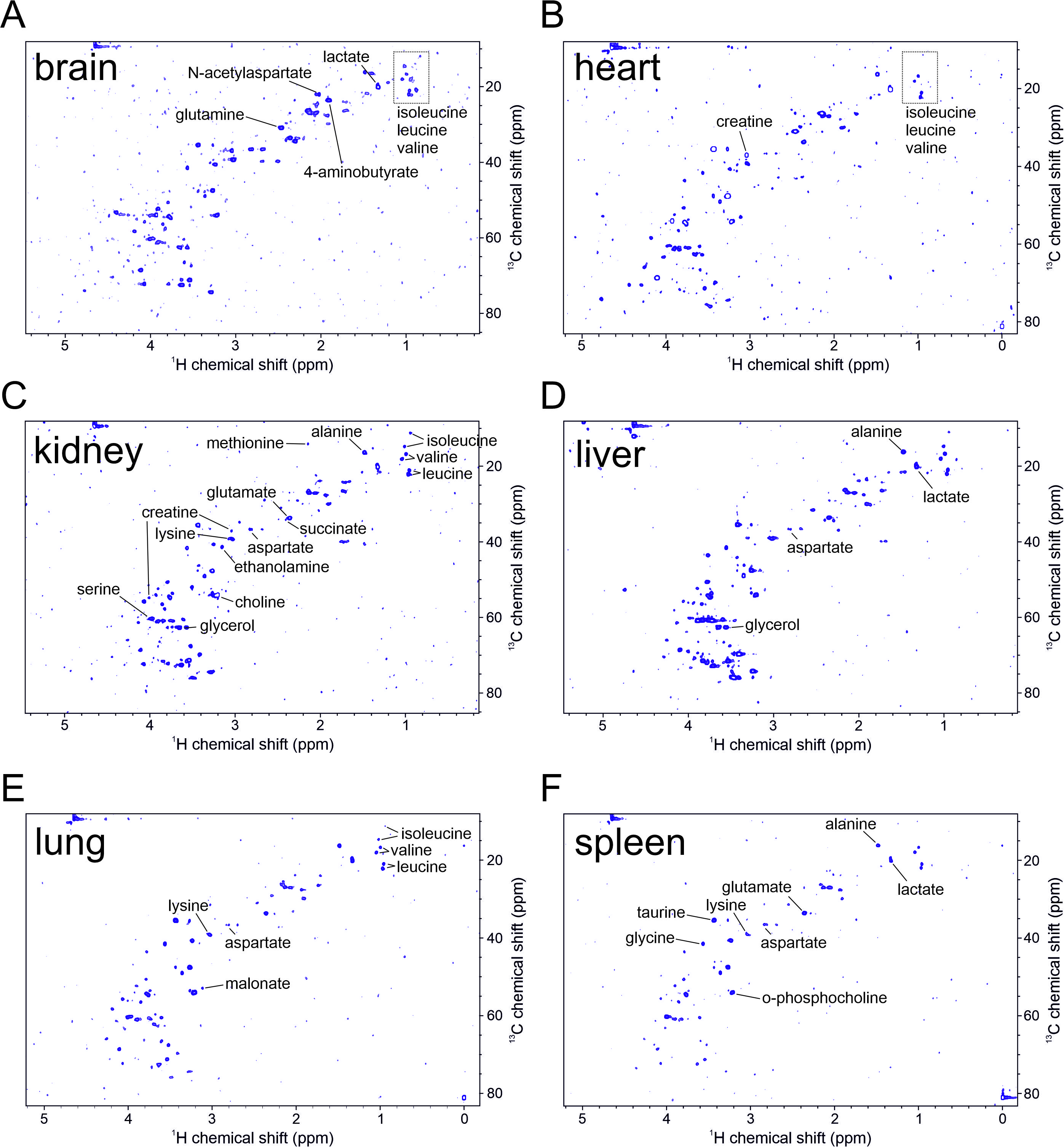

Supplement: Supplementary file 1 [file biomolecules-11-00235-s001.zip › Biomolecules 1047494/SFig_2_HSQC.jpg]
